# Rehabilitation and palliative care: histories, dialectics and challenges

**DOI:** 10.12688/wellcomeopenres.16979.1

**Published:** 2021-07-02

**Authors:** Helle Timm, Jette Thuesen, David Clark

**Affiliations:** 1 National Institute of Public Health, University of Southern Denmark, Copenhagen, 1455, Denmark; 2 REHPA, the Danish Knowledge Centre for Rehabilitation and Palliative Care, Odense University Hospital and University of Southern Denmark, Nyborg, 5800, Denmark; 3 School of Interdisciplinary Studies, University of Glasgow, Dumfries, Scotland, DG2 OXY, UK

**Keywords:** Rehabilitation, Palliative Care, Combined Rehabilitation and Palliative Care, care specialties, critical perspectives

## Abstract

Rehabilitation and palliative care are health care fields with separate histories but some recent convergences. Both have been identified as components within universal health coverage and each is the subject of a supportive World Health Assembly Resolution. We draw on the historiography of the two specialties, a recent systematic review of their engagement with each other as described in 62 studies, and critical policy perspectives to examine how rehabilitation and palliative care have been framed as potential partners in care. We examine the changing patient groups served by each field and the organizational forms that combined rehabilitation and palliative care (CRPC) may take. We explore the implications of such collaboration for the underlying goals and values of the two specialties, where each is the subject of changing definitions with differing responsibilities for regulating access to services as well as assuring and documenting quality. We conclude that to be effective CRPC must adapt to the highly segmented and specialized systems in which it is required to operate, recognizing that rehabilitation and palliative care are themselves co-constructors of such segmentation and specialization, but also potential agents for change.

## Introduction

Rehabilitation and palliative care emerged as fields within modern healthcare during the course of the 20th century. Their histories have been mainly separate, asynchronous and unconnected. Rehabilitation as a field gained traction after World War I and was initially focused on military personnel returning from conflict ^1^. Palliation as a field emerged in the decade after world war two and concentrated at first on those who had lost the ‘battle’ against cancer ^2, 3^. Beyond these militaristic dimensions, they seemed to have little in common.

As they have evolved, however, both rehabilitation and palliation have encountered shared milestones and benchmarks. Both are characterized as interdisciplinary fields and have coalesced around professional societies and groups to build capacity and promote their work. Both have been defined by the World Health Organization in ways that have enhanced their significance and credibility. Each has sought, with some difficulty, to build an evidence base to underpin practice. Both have had to engage with a changing ‘target group’ of people who might benefit from their services. Both have quality of life as an over-arching goal. Each can be seen within the architecture of current health care policy.

In most recent times there has been evidence of connections between rehabilitation and palliation in relation to people with significant burdens of illness, whether or not death is expected. A measure of dialogue has developed between the two specialties. Collaborations have begun that seek to forge joint approaches to service delivery and clinical care. The conceptual language of the two fields has found points of overlap leading to new terminologies and ‘framings’. Whilst these endeavours are in the main still at an early stage, we believe they merit examination, and as we shall see, there is a body of literature, which now illustrates the encounter between rehabilitation and palliative care. In this paper, we place these collaborative endeavours in a historical and societal context, we explore the evidence on how and using which arguments rehabilitation and palliative care are combining, then, using a more critical lens, we go on to consider the circumstances and perspectives that are shaping their mutual development.

In attending to the latter, we are inspired by a post-structural approach to policy analysis, including a practical guide that asks and seeks to answer specific questions concerning the representation of any particular ‘problem’ in the policy context ^4, 5^. We therefore examine the ‘problems’ that have been described in order to make an argument for rehabilitation and for palliative care and the emergent argument for bringing the two fields together in combined rehabilitation and palliative care (CRPC).

If rehabilitation focuses on functional capability, palliative care attends to the relief of suffering. They do this through differing frameworks, guidelines and practices. Each envisages a contrasting view of the person who can benefit from their offering. Yet as the ageing population exhibits greater co-morbidity and complexity of health and social care needs, it may well be that rehabilitation and palliation are finding themselves in a shared space. We conclude by suggesting that there might be benefit to be gained in a closer alignment between the two, but we argue that both must begin to forge an understanding of shared societal circumstances, a common language and mode of practice that is most relevant to contemporary needs and policy contexts, and endeavour to be less aligned to institutional histories and the boundaries they impose. As part of that, we argue some contradictions, worries and ‘silences’ have to be addressed.

To summarize, in rehabilitation and palliative care we have two fields, each with distinct longer histories of their own development. Mostly these exist in parallel and are unconnected to one another. Recently there have been some points of convergence but these are still only lightly sketched. Whilst the two fields are substantively different, they do share certain similarities in accounts of their development. Historical writing about each has increased in recent decades, but we are not aware of any work that seeks to contrast and compare the two fields and their histories in ways that lead to a critical post-structural point of view, which is our purpose here.

## Aim, sources and analytical frame

The aims of this paper are: 1) to explicate the separate histories of rehabilitation and palliative care, paying attention to roots, definitions and core concepts; 2) to explore what is known about the policy and practice issues involved in bringing the two together; and 3) to offer a critical analysis of current arrangements and a perspective on future development.

In doing so our sources derive from: 1) the historiography of palliative care and rehabilitation; 2) a literature review of the arguments for and the documentation relating to combining the two fields; and 3) inspiration from a post-structural perspective when making sense of rehabilitation and palliative care within areas of health policy.

Following Bacchi (4) and Bacchi and Goodwin (5), we ask: 1) What is the ‘problem’ represented to be? 2) What presumptions or assumptions underlie it? 3) How has this representation come about? 4) What is left unproblematic within it, including silences and differing representations? 5) What effects are produced by this representation of the ‘problem’? 6) How/where is the ‘problem’ produced, disseminated and defended?

## Histories of rehabilitation and palliative care

The process of identifying the historical roots of the field is a feature of commentaries on both rehabilitation and palliation, and often begins with the etymology of the key terms in use. Both fields generate a discourse which seeks to establish the work as having a long history, and not simply as a specialty of the modern period. Some scholars comment on references to what is later termed palliation or rehabilitation that can be found within medical treatises published from the mediaeval period onwards.

Rehabilitation tends to emphasise its linguistic origins as deriving from the medieval Latin term *habilitatus/habilitar*e, meaning ‘make fit’ or ‘enable’ ^6^. American medical literature has associated rehabilitation therapy with the Hebrew term *refusa*, meaning healing ^7^. Here the ancient roots of the rehabilitation approach are also given prominence – early Chinese movement practices to relieve pain, 2nd century Galenic interventions to ameliorate military injury, and 5th century Greek exercises for the prevention and treatment of disease. LePlege and colleagues show how this is linked to the distinction between able-bodied and disabled people, that began to assert itself in the 14 ^th^ century, after the Black Death ^8^. By the 18 ^th^ century, Enlightenment assumptions about the ‘natural order’ of disability were beginning to be challenged, opening the door to new approaches with rehabilitative intent. Special institutions emerged to treat those with sensory impairments and by the 20 ^th^ century, to treat disabilities of all kinds. Now medicine, social reform and educational interventions developed so called ‘restorative practices’ that combined to ameliorate impairment and disability across a broad spectrum ^9^. Then in the aftermath of World War I, social programmes to support those wounded and injured in conflict began to focus not just on treatment, but also developed a strong imperative to return disabled servicemen to the workplace, further deepening the moral dimensions associated with disability, re-enabling and rehabilitation.

For palliative care, Stolberg asserts that ‘palliative care is definitely not an invention of the nineteenth or twentieth century’ ^10^. He sees evidence of interest in these matters from the end of the Middle Ages and shows that by the end of the sixteenth century medical writings using terms like *cura palliativa* and *euthanasia medicinalis* were well established and having an influence on practice. Stolberg points out that in the early modern period (c15-1800), physicians were significantly engaged in discussion about their human duty to support and care for those whose conditions were clearly incurable. This related in particular to cancer, tuberculosis (phthisis), and ‘dropsy’, which included heart or liver failure and kidney disease.

Numerous writers from within palliative care refer to words like *palliare* and *pallium* in their discussions of early origins ^11^. Here the notion is that palliation is a last resort when all curative measures have failed. Cloaking or shielding the patient from suffering is therefore the goal when death is inevitable or imminent. The desired outcome is then the ‘good death’, often described by European writers in the 19 ^th^ century as ‘euthanasia’ and seen as a science that controls the oppressive features of illness, relieves pain, and ‘renders the supreme and inescapable hour a most peaceful one’ ^12^. 

### Key individuals, professional groups and knowledge claims

For both fields, as the formalisation of basic principles was elaborated, associated with the relief of pain and suffering in the face of mortal illness, or with actions likely to re-enable, promote recovery and enhance physical wellbeing, so we also see the emergence of key individuals who through their writings, teaching and innovative clinical practice, start to influence wider thinking and practice. From this later period and into the 20 ^th^ century there is an emerging celebration of foundational individuals, mainly medical doctors, who began to take a more focussed interest in palliation and rehabilitation. These include the authors of specialist theses, textbooks and even key research articles in medical journals. We thus see how each field also defines itself in relation to oft-repeated biographical narratives that shape its unfolding discourse.

For palliative care, some later Victorian writers on the care of the dying can be seen reaching a wide audience. Most famous among these is perhaps William Osler, who conducted a study of ‘the act of dying’ among 486 patients at the Johns Hopkins Hospital between the years 1900–1904 ^13^. He claimed that only one fifth of his patients suffered physical, mental or spiritual discomforts and maintained that for most ‘death was a sleep and a forgetting’. Of more specific relevance, in so far as his treatise laid out a clear set of prescriptions for the care of the dying, including pain and symptom management, dealing with wider distress, diet, and the organisation of the sick room, is William Munk. His short text entitled *Euthanasia: Or, Medical Treatment in Aid of an Easy Death*, published in 1887, gained favourable reviews on both sides of the Atlantic and Munk has been described as the grandfather of modern palliative care ^12^. Alfred Worcester’s short book, entitled *The Care of the Aged, the Dying, and the Dead,* written for an American audience, highlighted the diminishing interest of doctors in care for the dying patient and drew on his own practical wisdom about clinical aspects of the ‘process of dying’, its associated symptoms, the role of fluids, and the problem of restlessness ^14^. He attends to the environment of the dying person’s room, to the need for light and for ventilation and endorses the liberal use of opiates, considering morphine to have ‘no rival’. He deals with the role of faith and religion, with patients’ visions and hallucinations, and with the question of uncertainty. His work is often seen as foundational to the field of modern geriatrics, but undoubtedly, he is influential in subsequent thinking in palliative care.

Certainly, Osler and Worcester had an influence upon Cicely Saunders from the 1950s as she began to formulate her ideas about the care of the dying. The life and legacy of Dr Cicely Saunders has been documented in detail ^15^. Trained as a nurse, social worker and physician, from 1958 she began writing about the need to improve care of the dying and in 1967 established St Christopher’s, the world’s first modern hospice, with a commitment to teaching and research, as well as clinical care. A hospice movement, peppered with other charismatic leaders, then began to develop in many countries, often using variants of the St Christopher’s approach adapted to local conditions, and achieving growing recognition for what was to become a new field of medical and healthcare practice, and which by the mid-1970s, came to be known as palliative care.

One of the earliest groups to form around the rehabilitation field was the German Society of Physical Medicine and Rehabilitation, established in 1878. The term ‘Physical Medicine’, rather than Physiotherapy, was first used by the London Hospital in England in 1921 ^16^. Modern rehabilitation has several origins, however. In Europe, Robert Jones has been described as the founding father of modern orthopaedics and rehabilitation ^17^. With a background in surgery and orthopaedics, Jones developed the treatment and training of crippled children together with the nurse Agnes Hunt at Basechurch in Shropshire, UK. In World War I he joined the conflict as a Captain in the Army Reserve. At the frontlines, he noticed that the treatment for fractures, in war as in domestic hospitals, was insufficient. His efforts to promote better rehabilitation of wounded soldiers led to the establishment of specialized military hospitals where he was assisted by American surgeons. At the end of his career Jones dedicated his time to creating wider social acceptance for people with disabilities, who were highly stigmatised, and helped facilitate the recovery of soldiers to reintegrate them back into society.

Also from the UK, Marjorie Warren has been described as the ‘mother of geriatrics’ and a pioneer in introducing rehabilitation medicine into the care of older people, hemiplegics and amputees ^18^. Like Jones, Warren’s early career was in surgery. In 1935 it took a dramatic turn, when the nearby Poor Law Infirmary was annexed into the West Middlesex Hospital where she was Deputy Medical Superintendent. The majority of the new patients were chronically bed-ridden and many were labelled ‘incurable’ in view of their musculoskeletal or central nervous system handicap. Warren personally introduced a systematic assessment of all patients and a multidisciplinary rehabilitative approach. ‘To be successful, rehabilitation must be undertaken by everybody coming in contact with the patient, beginning with the gate porter’, she said, and added that ‘underlying that approach is a very profound philosophy, which we should do well to adopt’ ( *ibid,* p.255). At the time this represented a deeply altered way of thinking about chronic care, and of impairment in old age - seen as something malleable. As Gilleard and Higgs show in commenting on Warren’s work: ‘By “active” rehabilitation, old age was to be rescued from the margins of society and, through the agency of “hospital” medicine, returned to a real and valued position within society’ ^19^.

The origins of modern rehabilitation are also linked to the role of Frank Krusen, the American physician who, after contracting tuberculosis in 1922, became interested in the process of recovery. His focus was on physical medicine, which he established in a programme of physical therapy and inpatient rehabilitation at Temple University in 1929 ^20^. Nine years later, following the efforts of Krusen and colleagues and with the support of philanthropic funding, the American Society of Physical Therapy Physicians was established, later becoming the American Society of Physical Medicine and Rehabilitation. The International Federation of Physical Medicine and Rehabilitation was founded in 1950, and by 2020, renamed as the International Society of Physical and Rehabilitative Medicine, had 135,000 members from 75 countries ^21^. Parallel to the development of the Society another important organisation developed, also with its origins in the USA. What is now known as Rehabilitation International was founded in 1922 under the name The International Society for Crippled Children by the social worker Bell Greve and the orthopaedic surgeon Henry Kessler; it had the explicit aim of giving people with disabilities the chance to lead full and productive lives ^22^. These two organisations illustrate a tension in modern rehabilitation between a medical/clinical and a social/political focus which is also founded in explanatory models of disability ^8^.

In 1988, the European Association for Palliative Care (EAPC) was formed in Milan, Italy, and Vittorio Ventafridda, who had been involved in the early discussions and shaping of the World Health Organization (WHO) approach to cancer pain relief, became its first president the following year ^23^. Other regional associations followed with the Latin American Association of Palliative Care (2000), The Asia Pacific Palliative Care Network (2001), the African Palliative Care Association (2004). In 2003 the first ‘summit’ on international palliative care development took place in The Hague. Others followed in Seoul (2005), Nairobi (2007) and Vienna (2009) – leading that year to the creation of the Worldwide Palliative Care Alliance ^24^.

For both fields – rehabilitation and palliative care - the processes of specialist and policy formalisation begin with the coalescence of ideas, practices, knowledge claims and sites of innovation. This involves the formation of a habitus that will subsequently provide a platform from which to pursue technical and bureaucratic strategies that will facilitate formal accreditation for practitioners and recognition in policy arenas. In this the two fields are not co-terminous. The habitus of modern rehabilitation starts to be formed in the years after World War I, whilst the habitus of modern palliation is largely a product of the period after World War II.

Stiker maintains that central to the rise of rehabilitation as a modern field of practice is the goal of transforming the disabled body in ways that will allow it to fully enter the realm of material production and consumption ^25^. People with physical differences are exposed to rehabilitative practices in order that they can then disappear from discourse and become assimilated into a world where only the able bodied have agency. In this scenario the individual body, rather than the social world is seen as the arena for change. It is a normative orientation centred on compliance.

In palliation, the central organising concept is that of ‘total pain’, first described by Cicely Saunders in the early 1960s ^26^. It recognises the multi-facetted nature of pain as suffering, which can comprise physical, mental, material, social and spiritual dimensions. Once described as both a nomenclature of inscription and a nomenclature of facilitation, total pain defines the goals of the modern palliative project – to relieve suffering at the end of life using multi-disciplinary approaches and technologies. It too requires a measure of compliance, including accepting the use of powerful pain-relieving medications that are often demonised in other contexts, being willing to engage in end of life conversations, life review, the reconciliation of troubled relationships, and the acceptance of death.

In the second half of the 20 ^th^ century, both fields sought specialist recognition of these specific knowledge claims and practices. For rehabilitation this was first achieved in the USA in 1947, when ‘physical medicine and rehabilitation’ was formally recognised by an independent Board, established under the authority of the American Board of Medical Specialties ^27^. Forty years later, in 1987, a specialist training in palliative medicine was first authorised by the Royal Colleges in the UK. In the next two decades more than 20 other countries took a similar path; often, as in the USA in 2007, recognising palliative medicine as a sub-specialty of some other field, such as oncology, anaesthetics or cardiology ^28^.

### Policy recognition and changing definitions

Within these processes of recognition, both rehabilitation and palliative care became the subject of WHO policy documents that in each case define the field of activity and locate it within a discourse of health system planning and resourcing.

As early as 1972, WHO produced its *International Classification of Impairments, Disabilities and Handicaps* ^29^. But as LePLege and colleagues show, this received mounting criticism from disability groups that pointed to the absence of reference to the social determinants of disability ^8^. This was acknowledged in the foreword to the fourth edition of the classification in 1993 and led to the *International Classification of Functioning, Disability and Health* (ICF) in 2001. In turn, the WHO engagement with palliative care came through the route of addressing the global problem of cancer pain relief, in a key 1986 publication ^30^. This led on to subsequent work at WHO to shape and define the emerging field of palliative care, and in time to address its particular relevance to population sub-groups, such as children and older people ^31^.

Notable for both fields, however, is that each became the subject of a World Health Assembly ‘resolution’, calling the WHO member states to action. For rehabilitation this came in the resolution on disability in 2005 ^32^. For palliation it occurred in the resolution on palliative care in 2014 ^33^.

The 2005 disability resolution drew on elements from a United Nations ruling, going back to 1993 and specifying that ‘States should ensure the provision of rehabilitation services to people with disabilities in order for them to reach and sustain their optimum level of independence and functioning’. The resolution was in turn given further support by the *United Nations Convention on the Rights of Persons with Disabilities*, which came into force in 2008. This document, known as the CRPD, states in article 26 that rehabilitation services must begin at the earliest possible stage, should be based on multi-disciplinary assessment and should include the provision of assistive devices and technologies. In 2011, WHO laid out a process for the production of guidelines on ‘health-related’ rehabilitation. The motivations for this included: poverty reduction; a growing population that could benefit from rehabilitation; gaps in provision, access to and quality of rehabilitation; and the need to strengthen services within existing systems. In the same year the WHO World Report on Disability defined rehabilitation as ‘a set of measures that assist individuals who experience, or are likely to experience, disability to achieve and maintain optimal functioning in interaction with their environments’ ^34^. In 2017 WHO reinforced its commitment in this area, in a report with recommendations on *Rehabilitation in Health Systems* ^35^. The document makes the case for a greater focus on rehabilitation, in the context of ageing populations around the world, and with a particular need for development in low- and middle-income countries. The key recommendation is for rehabilitation to be integrated within health systems, with the ministry of health responsible for this at country level, thereby ensuring more rational and appropriate governance. This should in turn guarantee that rehabilitation contributes to the provision of person-centred care, across the care continuum. Rehabilitation should also be seen as part of universal health coverage, and by implication not an ‘extra’. Efforts should therefore be made to provide quality, accessible and affordable services to meet needs that have been assessed for specific populations. The document makes considerable strides to match attention to the widely used indicators of mortality and morbidity, with a third dimension, that of functioning, and is the cornerstone to the WHO Rehabilitation 2030 initiative. One global estimate suggests that 2.41 billion individuals have conditions that would benefit from rehabilitation ^36^.

The 2011 WHO definition of rehabilitation has been criticised for being too narrow, in describing rehabilitation as ‘a set of measures’ ^37^. A review of rehabilitation definitions, however, found no specific means or interventions which could define rehabilitation and found that rehabilitation has been variously described as ‘a set of measures’, ‘a process’ and ‘a health strategy’ ^38^. A new definition is being prepared in the Cochrane Rehabilitation group, which issued a provisional wording in October 2020 ^39^: Rehabilitation is a “multimodal person-centred process including functioning interventions targeting body functions, and/or activities and participation, and/ or the interaction with the environment” (..) aimed at “optimising functioning” (..) in (1) persons with health conditions (a) experiencing disability or (b) likely to experience disability, and/or (2) persons with disability” (p659). 

The first major WHO milestone, specifically oriented to palliative care, rather than the narrower field of cancer pain relief, occurred in 1990 ^40^. It came in the form of a technical report that considered more broadly what could—and should—be done to comfort patients suffering from the distressing symptoms of advanced malignant disease and also marshalled arguments for palliative care based on the magnitude of unrelieved suffering experienced by the majority of terminally ill people. Although methods for the relief of pain continued to be emphasized, other physical, psychological, social and spiritual needs for comfort were also included in the report's recommendations. This conceptualisation of palliative care turned on its concern with quality of life and comfort before death, emphasising the family as the unit of care, dependence on teamwork, and its relationship to curative interventions. It framed palliative care as: ‘… the active total care of patients whose disease is not responsive to curative treatment’, described the goal of palliative care as achievement of the best quality of life for patients and their families, and saw many aspects of palliative care as applicable earlier in the course of the illness in conjunction with anti-cancer treatment. 

Twelve years later, in 2002, a new definition of palliative care appeared from the WHO ^41^. Globally, the field was becoming better known to policy makers and practitioners, debates about its mission and scope were proliferating, and there was an increasing interest in expanding its vision beyond terminal care and oncology to make it available to patients and families where diseases of various types were not so far progressed, or where the distinction between curative and palliative approaches might not be so clear cut. Published in 2002, the second WHO definition saw some critical changes and is summarised as: ‘Palliative care is an approach that improves the quality of life of patients and their families facing the problems associated with life-threatening illness, through the prevention and relief of suffering by means of early identification and impeccable assessment and treatment of pain and other problems, physical, psychosocial, and spiritual’. 

Despite, this growing interest, it was not until 2014 that the World Health Assembly drew attention to the need for action on the limited availability of palliative care in most of the world, the avoidable suffering of millions of people and their families, and the need to create or strengthen health systems that include palliative care as an integral component of treatment.

The 2002 definition of palliative care is still the WHO position today. But in 2017 a *Lancet Commission Report on Pain and Palliative Care* introducing the phrase ‘serious health related suffering’ (SHRS), declared this to be the problem on which palliative care should be focussed, and stated that over 40 million people experienced SHRS in 2015, of which 25.5 million died ^42^. By this means, considerable expansion took place in the ‘target group’ of people who might benefit from palliative care, many of whom may not be imminently dying. The following year the International Association for Hospice and Palliative Care proposed a new definition and formalised it in a publication of 2020: ‘Palliative Care is the active holistic care of individuals across all ages with serious health-related suffering. Suffering is health related when it is associated with illness or injury of any kind. Health-related suffering is serious when it cannot be relieved without medical intervention and when it compromises physical, social, spiritual, and/or emotional functioning … because of severe illness (high risk of mortality, negatively impacts quality of life and daily function), and/or is burdensome in symptoms, treatments, or caregiver stress … and especially of those near the end of life. It aims to improve the quality of life of patients, their families, and their caregivers’ ^43^.

Through these diverse vectors of clinical research and reflection, the growth of professional societies, the focus on definitional matters and the engagement of non-state international actors in policy formation, we see that each field also seems to have a wider relationship to a deeper societal reality.

### Each field in social context

For rehabilitation, the close relationship is with disability. Stiker’s classic work uses the social construction and semiotics of disability to reveal how the condition has shifted from something that is seen as innate, fixed and natural in some people, to one that can be construed as temporary and malleable ^25^. His project seeks to uncover how the ‘difference’ of disability was over-valorised in earlier periods, but under-valorised in the modern ‘age of rehabilitation’, in a context where rehabilitation is co-extensive with pluralised disabilities and their extension in time.

For palliation the key relationship is with death. In the earliest writings, palliation is seen as a means to relieve suffering when death is inevitable and imminent. It is essentially focussed on the deathbed and its medical management. In its more recent manifestations, palliation is seen to have a wider role, located within a context of prognostic uncertainty and where the promotion of quality of life and wellbeing in the face of serious illness is the primary goal. This means, at least in relation to claims made for the modern field, that palliation should be introduced earlier in the trajectory of disease, accompanying and not displacing curative measures, and should also be available to those with chronic and complex conditions, where there may be no singular diagnostic starting point to a disease trajectory that is subsequently extended and erratic in its course. In this context, notions of ‘terminal care’ are played down and the scope of palliative care is widened.

So both fields relate to cultural understandings associated with stigma, inclusion and integration, functionality, fear, suffering and pain. They also respond to and in turn they shape and influence the changing perceptions of important clinical framings of ‘disability’ and ‘terminal illness’. As we shall see, it is in the inter-relations with these themes that each field seeks to generate new, progressive, even transformative narratives. That in turn has provided the conditions of possibility for them to come together in a new collaborative sub-field of practice.

In part we can see the two fields dialectically. They appear as opposite forces, engaged in different struggles. Rehabilitation seeks to triumph over adversity, chronic illness and disability, thereby opening up new life possibilities. Palliation seeks to relieve suffering, not to cure or modify its underlying cause, and in so doing it aims to bring about acceptance and short-term quality of life improvement in the face of inevitable death. We can see, however, some resolution of this dialectic. New treatment modalities, palliative interventions that extend life, innovative health technologies and an ageing population in which significant numbers of people experience multiple or co-morbidities, are all blurring the boundaries between the two fields and the target groups to which they are orientated. What then, we might ask, are the opportunities that result from a closer and more collaborative encounter between the two specialties? Thinking in this way the dialectic starts to be resolved and actors begin to see new forms of innovative practice that flow from mutual engagement.

## Combining rehabilitation and palliative care

Until very recently, in the key rehabilitation policy documents from WHO, the issue of living with a life-threatening disease and the need for palliative care was not addressed. Likewise, there were initially no references to rehabilitation in the palliative care documents. The key linking point that brings the two together is around the notion of universal health coverage. WHO states that ’Universal health coverage is defined as ensuring that all people have access to needed health services (including prevention, promotion, treatment, rehabilitation and palliation) of sufficient quality to be effective while also ensuring that the use of these services does not expose the user to financial hardship’ ^44^. For these reasons, universal health coverage has become a major goal for health reform in many countries and a priority objective of WHO. In so doing it has linked rehabilitation and palliative care in a continuum of care, and by extension fostered dialogue between them.

A reflective and seemingly landmark paper by Hockley from 1993 explores this issue, long before it appears to have gained policy endorsement ^45^. It asks if rehabilitation in palliative care is asking the impossible and concludes to the contrary, by affirming the value of ‘palliative rehabilitation’. Acknowledging the apparent contradiction between the two fields, Hockley draws on earlier work, mainly from the oncology setting which ventures to bring forward a connection between the two. In particular she highlights the work of Dietz, who argued that the goal of rehabilitation for people with cancer is to improve quality of life for maximum productivity with minimum dependence, regardless of life expectancy ^46^. For Dietz, there were four modes of cancer rehabilitation: preventative, restorative, supportive and palliative. Hockley takes the view that in palliative care, the goal of improving quality of life requires attention not only to physical symptoms but should also include ‘social, emotional and vocational’ dimensions: ‘Aligning rehabilitation to palliative care helps to sharpen the focus on these other aspects, integrating the expertise of multi-disciplinary professionals’ (p10).

In 2016 the Danish Knowledge Centre for Rehabilitation and Palliative Care published a narrative synthesis review of the literature concerning the arguments and the evidence for combining and coordinating rehabilitation and palliative care for people with life-threatening diseases including chronic obstructive pulmonary disease (COPD), stroke, cancer, and also for geriatric patients ^47^. A significant amount of content had emerged in the 23 years since the Hockley paper. The review covered publications in English, Swedish, Norwegian and Danish, published 2003–2013. Sixty-two articles were included, divided into four areas of focus: recommendations (29), patient perspectives (8), structure, organization, models and referral processes (36) and interventions (12). The literature was found to be of varying quality, with an absence of control groups in the intervention studies and inadequate methodological reporting.

However, a narrative synthesis asking why, when, how and for whom rehabilitation and palliative care are combined, resulted in four themes, reflecting the arguments for bringing the two fields together, but at the same time exhibiting a lack of consensus on when and how rehabilitation should be offered, the relevance of rehabilitation to non-cancer conditions, and recommendations for co-ordinating rehabilitation and palliative care.

### Arguments for the co-ordination of rehabilitation and palliative care (why)

Whilst recognising the need to clarify the differences and similarities of practice in the two fields, these arguments included the recognition that patients may need both types of intervention, with appropriate coordination between them. The unmet ‘rehabilitative’ needs of people in palliative care (and vice versa) were promoted as arguments for combining the two. These unmet needs were often articulated by relatives. Mostly the needs for rehabilitation for those in palliative care comprised: the need to be normal and in control, the need for better every day functioning and mobility, and the need to alleviate the fear of being a burden. People in rehabilitation, however, might have unmet expectations that professionals should take up end-of-life questions, though some studies documented the aversion to having end-of-life conversations.

Another argument for combining the two approaches was that patients, relatives and professionals may have changing, conflicting and ambivalent preferences for rehabilitation and palliative care and these should be met by both perspectives, simultaneously. There was also awareness of unpredictability in the disease course for many patients in ways that can require ad hoc and co-operative solutions. This argument was particularly prominent in the context of COPD. This in turn raised organizational issues about coherent engagement between the two sets of services. Studies showed that professionals in the field of rehabilitation was unlikely to identify palliative care needs, and conversely. Finally, there was a poorly documented argument that coordinating rehabilitation and palliative care would maximise efficiency.

### Lack of consensus on when rehabilitation and palliative care should be offered and coordinated (when)

The review found no consensus on when in the continuum of care, rehabilitation and palliative care become relevant. With regard to the timing and coordination of rehabilitation and palliative care during the course of the disease, the review identified three models: dichotomic (rehabilitation and palliative care are offered in different phases); progressive/complementary (both rehabilitation and palliative care are introduced from the time of diagnosis, gradually shifting in emphasis from the former to the latter); and ad hoc/complementary (both rehabilitation and palliative care are introduced at the time of diagnosis and complement each other during the course of the disease, responding to the fluidity and changing manifestations of the disease). Yet the literature study showed that both the notion of a phased course of disease and the widely used idea of a sliding transition from rehabilitation to palliative care are contestable. The relationship between rehabilitation and palliative care varies across diagnosis and individual courses of disease, depending upon prognosis and the situation and preferences of the patient and his or her relatives. The transition from rehabilitation to palliative care can be challenging for patients, relatives and health care professionals. The idea of a breakpoint where the switch from ‘cure to care’ takes place is not well supported in clinical practice or in the illness experiences of people living with life threatening disease. The review did not support the notion that coordination of rehabilitation and palliative care is particularly relevant in the early course of the disease, nor is there evidence in support of the recommendations concerning when rehabilitation and palliative care should be coordinated. 

### Coordination of rehabilitation and palliative care is relevant for cancer patients and those with other conditions (for whom)

The literature review included patients with COPD, stroke, cancer and geriatric patients and found that coordination of rehabilitation and palliative care may be relevant for these groups of patients or conditions – as well as for other patient groups with progressive disease leading to death, though these were not included in the review.

Papers arguing that rehabilitation should be available across the entire illness trajectory, including the terminal phase, were found for all included target groups. Five papers were from cancer care, four papers from COPD, two papers were from geriatrics and two were from stroke. 

### Recommendations for how rehabilitation and palliative care can be coordinated (how)

The review showed that coordination of rehabilitation and palliative care can be organized in various ways. These include partly integrated units within which there is rehabilitation with palliative components, or palliative care with rehabilitation components, or alternatively in co-operating units where otherwise independent rehabilitation and palliative care teams work together. Rehabilitation components are predominantly represented by the integration of single professions (such as physiotherapy, occupational therapy and speech and language therapy) adopting a function focused approach. Palliative care components are predominantly addressed in terms of end-of-life conversations.

The review found no evidence of fully integrated units, where rehabilitation and palliative care are organised together in equal measure. It highlighted recommendations to establish effective coordination of rehabilitation and palliative care, including: systematic assessment procedures (such as screening, referral, needs assessment); individual plans and objectives which are revised frequently; an appropriate balance between autonomy, help and support; and outcome measures to establish effectiveness. Intervention studies on ‘palliative rehabilitation’ were considered as interventions that combined rehabilitation and palliative care. The limited evidence base in this area was noted, though a handful of studies did suggest that people with serious and progressing illnesses may benefit from rehabilitative interventions in the later stages of their disease. Five out six palliative rehabilitation intervention studies were about cancer.

From 2016 onwards, more publications have addressed rehabilitation and palliative care as coordinated efforts. A new literature review, by Thuesen *et al.* is now in process, still, not published, and preliminary results indicate an increase in palliative rehabilitation intervention studies (personal communications). A Danish study by Nottelmann *et al.* ^48^ for example, describes a palliative rehabilitation intervention model, for patients newly diagnosed with advanced cancer, and presents data on how it was utilised during a randomised control trial. In Portugal, another review is underway on rehabilitation interventions in palliative care ^49^. Its protocol states that ‘Both palliative care and rehabilitation share essential characteristics in that they are symptom-oriented approaches that focus on function and comfort within a holistic framework. The goal is to promote independence in self-care activities, better symptomatic control, and stabilization of functional decline in line with individual life preferences. Ongoing assessment of patient response indicating improvement, stabilization, or deterioration is conducted, and regimens are modified as appropriate’ (p.2350). 

Preliminary findings from the Portuguese study ^49^ seem to support those from the 2016 review and indicate that more interventions are being developed, mostly in the form of ‘palliative rehabilitation’. They also seem to indicate that social integration and inclusion in society is not mentioned as a goal when rehabilitation encounters palliative care. Rather, functioning as a goal is described in terms of basic activities of daily living and mobility. It appears that, when rehabilitation is described within a palliative care context, the goal of rehabilitation is narrowed down.

Based on the above explorations, it is possible to draw out some analytical similarities and differences between the core elements of rehabilitation and palliative care ( Table 1).

**Table 1.** Contrasting and comparing the elements of rehabilitation and palliative care.

	Rehabilitation	Palliative care
**History and key relationships**	World War I. Disablement and disability	The Cold War. Cancer and death
**Focus area and concepts**	Functionability, coping ability, hope of normality and inclusion in society, ICF	Relief of suffering, hope of meaningfulness in the history of life and in everyday life, total pain
**Perspective**	Short- and long-term goals, function Development or active maintenance Control Doing and becoming	Mainly shorts-term goals Death as a natural process Relatedness Being
**Norms**	Norm of activity – to contribute, take part	Passiveness is legitimized; to receive/to draw back
**Expanded target groups**	From disabled persons to ‘not yet disabled persons’ in terms of health conditions more broadly spoken Expanding to advanced stages of the disease	From terminal ill cancer patients to all persons suffering from life threatening illness + their relatives Expanding to earlier stages of the disease
**Compliant to**	Goal setting and functionality Discourse of recovery The individual body as the arena for change	End-of-life conversations, the acceptance of death etc.

## Critical reflections

Our account of the histories and more recent coming together of the fields of rehabilitation and palliative care raises a number of critical issues. It is helpful to situate these in their wider societal context. Both fields demonstrate some of the characteristics of healthcare delivery and governance in a changing policy context. In particular, we see them shaped by elements of public health and social care strategies that seek to regulate populations through cultural and normative inscriptions, for example concerning ‘reablement’, ‘autonomy’, ‘a good life’ and indeed ‘the good death’ ^50^. These state-initiated public health strategies are forged at the macro level of policy and government, organized and assessed at the meso level of health care authorities and professional bodies, but also resonate at the individual level in which they are actively internalised by citizens in society ^51^.

Using the perspective of Rose ^52^ and his conception of ‘the social state’ that dominated Western welfare governance in the first half of the 20 ^th^ century, we can see that citizens had to *be able to* work and provide for themselves and their families. For those who, for a while or good reason, were not able to do so, society was – ideally – there to help ‘from cradle to grave’.

During the late 1960s and into the 1970s as the need for and possibilities of welfare seemed endless and the economies of western societies did not, social movements as well as economic and political challenges to the social state model of welfare governance led to new strategies of public sector management ^53, 54^. Hospice care emerged in this context, growing out of charitable, third sector and non-profit organisations, often forging partnerships with state providers, and drawing attention to the social state’s failure to fully attend to care at the very end of life. 

During the 1980s and the 1990s, New Public Management (NPM) became the leading political strategy for governing western societies in an increasingly globalized world. Based on ideas about the construction of an effective public sector through a managed market orientation, internal competition, need assessment and outcome measurement ^55, 56^, NPM gained considerable currency. More recently, ideas about New Public Governance (NPG), with its concerns about the policy challenges involved in public welfare in late modern societies have also been influential ^57^.

A further neologism is that of the New Public Health (NPH), which emerged from the 1970s; focusing on the environment, health promotion, disease prevention – and risk ^58, 59^. The prevention of risk was (and still is) seen as mainly an individual responsibility, albeit supplemented by specialist guidance, laws, and regulations. From the 1980s health is therefore defined as the overall concept and the core resource to ensure a life of quality ^60^.

While NPG was from the beginning inspired by the claims of social movements concerning citizens’ rights, user involvement and the ownerships of one’s own body, it may now be more concerned with involving citizens and ‘users’ in securing quality of life and health and social care, across public, private and voluntary sectors and stakeholders.

As we have seen, the target groups of both rehabilitation and palliative care have changed and expanded over time and in these changing contexts. The two fields are now crossing over each other in a broader focus on lifestyle diseases, quality of life and the inheritance of a form of welfare thinking that endures across the life course, including health promotion, disease prevention, treatment, rehabilitation and palliative care for all ages and diseases. Crucially from the perspective of global health, this must be in the context of universal health coverage.

### Expansion of target groups, timespans and contexts

While the ‘problem’ underlying the introduction of rehabilitation was represented as disablement from war or disaster, the ‘problem’ through which palliative care was represented was that of unrelieved suffering and ‘total pain’, when dying from cancer. What then is the ‘problem’ today and can it be combined for the two fields? In our view the problem represented can be seen as *premature death from and lives spent with prolonged chronic diseases – and the lack of wellbeing and quality of life related to both, not least in the later stages of life*. These challenges are in turn framed as life-style symptoms and diseases.

The main causes of death in western societies are chronic diseases (cancer, heart and lung diseases) and many people live and die with associated co-morbidity. At the same time, through better life circumstances and improved treatment, average life expectancy is growing among western populations. Some diseased people live for longer, albeit with severe symptoms of an ‘un-healthy life-style’, several diseases, and late side effects from treatment. In this context, the population of older people with diminished functionality and in need of care will continue to grow for some time.

This understanding of the ‘problem’ within the two fields explored here leads to expanding target groups and expanded timespans for professional intervention. Rehabilitation may concern reablement after disasters, diseases, traumas, treatment, alcohol abuse and many more causes. The relief of ‘total pain’ may be relevant to people suffering from a severe disease or situation - from the time of diagnosis or the causative event, and right up to the time of death, and indeed beyond for the relatives concerned. The literature review discussed here confirms that the combination of rehabilitation and palliative care is relevant for all the groups included (people with COPD, stroke, cancer and geriatric diagnoses), and probably several more. It also points to the changing needs and preferences that occur during unpredictable and often prolonged disease trajectories, which in turn support arguments for combining the two approaches.

If health and quality of life are defined as the overall wished for outcomes of health interventions throughout the lifespan - where does this leave, or limit, rehabilitation and palliative care, and the combination of the two? As seen in the review, there is no evidence of when and where in the disease course a coordinated effort of rehabilitation and palliative care will be most efficient or effective, even though experienced as relevant.

While rehabilitation of soldiers after war, as well as relief of pain among dying cancer patients, was mainly a hospital effort, nowadays rehabilitation and palliative care are taking place in many different settings and contexts, such as in peoples’ private homes, in nursing homes, in hospices, in special institutions, in day care – and in hospitals. The target groups and contexts for rehabilitation and palliative care, and the two in combination, therefore seem to be expanding and may include many more people in many more types of organisational settings. As seen in the review, CRPC works within and across different organisational contexts.

### Control of admission to rehabilitation, palliative care and CRPC

WHO strategies for public health emphasize the empowerment and enablement of all citizens - defining health as the totality of physical, mental and social wellbeing ^61^. At the same time this comes with a heavy emphasis on individual responsibility in creating for oneself, not only a healthy life, but also a good death. Within the governance context we have outlined here, the ‘conduct of conduct’ becomes an important dimension, often realised through exhortatory instruments, an emphasis on compliance and the internalisation of personal responsibilities. These dimensions are of course at work within our two fields of concern – rehabilitation and palliative care.

One example is a study showing how Danish courses of rehabilitation among cancer patients contribute to the construction of a narrative of ‘being-as-if-well-again’ ^62^. Such ideas seem not too distant from the aim of ‘normalization’ for disabled soldiers returning from World War I. Research in English hospices has documented how ‘the good death’ is socially constructed and negotiated within a special kind of setting for death and dying, that is excluded from everyday life and for certain groups of dying people ^63^. Recent research in Danish hospices has documented how hospice care is continuously negotiated between the values of hospice philosophy and the demands of the public health care system in the context of its requirements for documentation, referral and discharge ^64^.

Another role for the relevant professionals in making the system work is in aligning rehabilitation, palliative care and now CRPC, with the dominant system logic of NPM. This promotes a focus on competition through quality assurance and the measurement of outcome. Here some of the instruments include documentation procedures, evidence-based practice, quality indicators, clinical guidelines, education, clinical trials and scientific publications. All of these can be seen co-shaping the inclusion and exclusion of patients, the changing roles of professionals, and the structures of organisational practice. Examples include the measurement of needs, goal-setting and quality of life using screening instruments and structured questionnaires, that in turn determine the right to admission or not, the professional effort to be deployed, and the success, or not, of this effort. When rehabilitation and palliative care expand to more extensive target groups and their scope becomes wider, control of admission and access then becomes critical. As described in the review, needs assessment becomes an issue in providing access to rehabilitation and palliative care. Needs is, however, a dynamic concept shaped by policy and resource contexts ^12, 65^. For example, when needs assessment was first introduced in the United Kingdom context in the 1980s, health authorities were instructed to refrain from assessing needs that could not be met ^66^. We now see rulings governing the number of hours of rehabilitation that should be made available to older people. Likewise, entry to hospice in Denmark is mainly for those imminently dying.

When rehabilitation and palliative care are distributed according to needs, then needs must be considered as negotiated in a social context. The relation between societal strategies, the logic of the health care system and the roles of professional groups and service users can thus be seen to frame the distribution, development, organisation and practice of rehabilitation, palliative care and CRCP. Yet these overall circumstances and their consequences often appear to be silenced within professional discourse, or obscured by the language of prioritisation, cost-effectiveness and efficiency.

### Silences, battles and shortcomings

It is well recognised that despite moves towards disciplinary collaboration, medicine remains the dominant health care profession in most systems and jurisdictions. Whilst, as we have seen, medicine and prominent individual doctors play an important role in the history of rehabilitation and palliative care, today the narrative tends to focus on interdisciplinarity and to downplay, or even silence, the dominant role of the medical profession in both fields. As Weisz and others have noted, medicine divided into a large number of specialties and sub-specialities during the 20 ^th^ century ^67^. Weisz sees this as an intellectual strategy that divides problems and people into smaller and more manageable groups, a form of ‘divide and conquer’. Part of this is the formation of specialties that are actively multi-professional in character, of which rehabilitation and palliative care are good examples. 

The specialties we describe here and their partial coalescence therefore represent clearly this form of sub-division and also a particular characterization of ‘problems to be solved’. At the same time, however, merging specialties together is likely to expose fundamental differences in the assumptions, knowledge claims and goals of each field. A recent illustration of this can be found in the Lancet Oncology Commission report on the integration of oncology and palliative care, which sees the former as ‘tumour directed’ and the latter as ‘host directed’ ^68^. Such an over-determined bio-medical distinction is a good example of the cost that results from trying to integrate specialties without addressing their ontological and epistemological foundations.

Both rehabilitation and palliative care are specialties that presume they are already including or are able to include the other ^69^. But there are also internal worries within each specialty, to the effect that the aim and focus of the other will under-mine and might do harm to their respective patient groups. One example is the worry that the normative focus on recovery, restoration and reablement found in rehabilitation may put pressure on people suffering from severe illness who are being cared for within the palliative care frame of reference (personal communications). Conversely, there is concern that the lack of attention to recovery and enablement within palliative care, may lead to loss of function and quality of life (personal communications). Moreover, when rehabilitation and palliative care are combined, both may be changed. As described in the review, when rehabilitation is integrated in palliative care, it may be transformed in favour of function-focused and one-dimensional needs, while the complexity and interrelation of needs may be more difficult to articulate and are therefore silenced.

The literature review suggests that actual coordination and combination in clinical practice may be rather sparse, for example, palliative care integrates some functionality focus or rehabilitation incorporates some end-of-life conversations. It seems obvious in this context that CRPC would not replace either of the two specialties, but at the same time those specialties will continue to face challenges concerning their knowledge claims, effectiveness and value to service users and to society.

### The lack of theoretical understanding/definitions

Rehabilitation and palliative care, like many specialties, appear to lack explicit theoretical definitions of their core concepts and models of practice. The need for theory and theoretical understandings in rehabilitation has been addressed more recently ^8^. One of the theoretical issues discussed in rehabilitation is the conceptualisation of disability. As we have seen, the ICF was promoted by WHO in 2001. It was seen as ‘the answer’ to the previous one-dimensional ICIDH model by expanding to include the biological, psychological and the social, and to practice by operationalizing the bio-psycho-social model within a classification that reflects the person as a body, a self and a social being. The model has had a great impact on thinking and practice in rehabilitation. It has, however, also been criticised for a lack of clarity in its theoretical foundations and empirical testing ^8^. Moreover, ICF has been criticized for not addressing temporality and dimensions of quality of life. In particular subjective quality of life is a key missing component of the ICF model. In addition, rehabilitation for older people (reablement) has been criticised for a lack of theoretical understanding of ageing ^70^.

Whilst the definitions of palliative care do not mention the concept of ‘total pain’ directly, it is at the core of all of them, as the co-thinking of ‘pain and other problems, physical, psychosocial and spiritual *’*. Saunders recognized that ‘Much of our total pain experience is composed of our mental reaction’ ^26^. Commentators have shown, however, that addressing total pain requires both the prevention of physical pain and attention to patients’ illness experiences. But research about total pain in clinical practice and policy is limited and total pain, despite its central place in the history of palliative care, remains remarkably under-researched, conceptually and empirically ^71^. Consequently, engaging with total pain in clinical practice lacks a secure theoretical and analytic foundation.

With such conceptual deficits apparent in both fields, it is evident that considerable work will be required to bring them together in a holistic and effective manner.

## Conclusion

Rehabilitation and palliative care have different narratives and patterns of development, but are seemingly getting closer to one another. Our assessment of the diverse histories of the two fields and the limited literature on how they are coalescing suggests the logic of collaboration is articulated mainly at the common-sense level. Careful studies of the effects of collaboration and deeper consideration of the conceptual aspects of the two fields working together are largely absent. Yet combining rehabilitation and palliative care approaches appears to make prima facie sense for patients and service users, as well as for professionals involved in trajectories of life-threatening diseases and vulnerabilities. It also resonates with current approaches to health care management and governance. We are enthusiastic about the benefits that might result from closer connections, but take the view that both could profitably attend more to their shared societal circumstances, if a common language and mode of practice is to emerge that is aligned to contemporary needs and robust enough to deal with the current policy landscape, including its opportunities and threats.

Analysing these developments from a critical theoretical position – seeing rehabilitation, palliative care and CRPC as parts of a public health strategy, and asking questions, concerning the ‘problems’, silences and struggles that are uncovered, brings new perspectives to the narrative. We conclude with two key points, First, rehabilitation, palliative care and CRPC are all located within a health care system which is highly segmented and specialized. This has limitations but has also created opportunities for formal recognition and for collaboration and merger. Second, rehabilitation, palliative care and CRPC are themselves elements in and co-constructors of the current dominant strategies of governance and self-governance in health care. They are both expanding fields, yet increasingly constrained by methods of prioritisation that constrain citizens’ access to them.

The interest in combining rehabilitation and palliative care is growing. We look forward to further studies which can throw light on the consequences and implications. At the same time, such work should not proceed without some deep reflection on the conceptualisation of rehabilitation and palliative care in the context of broadly neo-liberal health care systems. Such work will benefit each field individually, as well as in their efforts to collaborate and coalesce.

## Data availability

All data underlying the results are available as part of the article and no additional source data are required.
