# Supplementary material for: Rehabilitation and palliative care: histories, dialectics and challenges
Source: Wellcome Open Res. 2021 Jul 2;6:171. [Version 1] doi: 10.12688/wellcomeopenres.16979.1 (PMC8790711; doi:10.12688/wellcomeopenres.16979.1)
Supplement: Peer review history

# Peer review: Reviewer response for version 1

**DOI:** 10.21956/wellcomeopenres.18746.r47972

**Published:** 2022-02-11

**Authors:** Kathy Eagar

**Affiliations:** 1 Australian Health Services Research Institute, University of Wollongong, Wollongong, NSW, Australia; 2 Australasian Rehabilitation Outcomes Centre, University of Wollongong, Wollongong, NSW, Australia; 3 Palliative Care Outcomes Collaboration, University of Wollongong, Wollongong, NSW, Australia

This is a very interesting and thoughtful paper. It contains relevant information on the historical development of both specialties, synthesizes their philosophical underpinnings and beliefs and draws these together in a credible way.

 The paper sets out to examine the histories and possible future of two specialties – rehabilitation and palliative care. This is both a strength and a weakness. It is a strength as this makes the topic manageable. It is a weakness as it fails to consider other specialties with similar histories and philosophies. The two that specifically came to mind are geriatric medicine and pain medicine, both of which are well developed specialties in their own right. If there is a case to combine rehabilitation and palliative care, why not also include geriatric medicine and chronic pain management?

 The paper is more an exploration of ideas rather than a research article. Yet some of the ideas could be better defined. The paper suggests the concept of ‘combined rehabilitation and palliative care’ without spelling out what a ‘combined’ service would look like. Other terms (including ‘collaboration’, ‘joint approaches, ‘alignment’, ‘integration’, ‘coordination’) tend to be used as though they are interchangeable with the idea of ‘combined’ service.  

 The key scoping issue in this paper is that it is, by design, theoretical rather than practical. This necessarily raises what I would call the ‘so-what?’ question. So what if palliative care and rehabilitation share some commonalities? Has the case been made that efforts should be made to bring them together as a combined service? If so how? These questions are not explored in this paper but suggest critical areas for further research and analysis.

 Australia (like the United States and elsewhere) has come to this issue from a practical perspective that forms a useful contrast to the theoretical framework in this paper. Australia brought palliative care and rehabilitation at a practical level in 1992. It did so by differentiating three types of hospital-based care – acute, subacute and non-acute care. Palliative care and rehabilitation were brought together as subacute care and this remains the case 30 years later.

 Subacute care has different data collections and funding arrangements to acute care.

 The separate introduction of a subacute care stream in Australia was driven by a decision to introduce the Diagnosis-Related Group (DRG) classification (originally developed in the USA) and to use it as the basis of hospital funding. This required that the scope of the DRG system be defined.

 The term ‘subacute care’ was coined for use in Australia in 1992 to describe care that should not be classified and funded by DRG. This was the defining characteristic that brought rehabilitation and palliative care together. They were not ‘acute care’ and should not be funded by DRG.

 Subacute care in Australia is defined as care provided for a person who requires health services but whose principal medical diagnosis (modified for factors such as age and procedures) is not adequate in explaining the need for, or the cost of, the services that s/he receives. In subacute (function-related) care the predominant goal is the enhancement of a patient's quality of life and/or improvement in his or her functional status. All rehabilitation, all palliative care, some mental health and some aged care are included in the definition of Australian subacute care.

 The reason I mention the Australian experience is to suggest areas for further research that arise from this paper. While the authors rightly caution on the need to reflect on the conceptualization of rehabilitation and palliative care in the context of neo-liberal health care systems, they essentially conclude that combining rehabilitation and palliative care is fundamentally a good idea. If so, the issue that now needs to be explored is how this is best achieved. This includes the levers for achieving better integration, how potential risks are managed, workforce implications and models of care.

 Our Australian experience is that the funding model has driven the two together without any serious detriments to either of them. Yet, even 30 years later, it is not clear whether other potential benefits have been realized. These issues remain ripe areas for further research.

Is the work clearly and accurately presented and does it cite the current literature?

Yes

If applicable, is the statistical analysis and its interpretation appropriate?

Not applicable

Are all the source data underlying the results available to ensure full reproducibility?

No source data required

Is the study design appropriate and is the work technically sound?

Yes

Are the conclusions drawn adequately supported by the results?

Yes

Are sufficient details of methods and analysis provided to allow replication by others?

Yes

Reviewer Expertise:

Health services research, palliative care, rehabilitation

I confirm that I have read this submission and believe that I have an appropriate level of expertise to confirm that it is of an acceptable scientific standard.

# Peer review: Reviewer response for version 1

**DOI:** 10.21956/wellcomeopenres.18746.r47964

**Published:** 2022-01-25

**Authors:** Leeroy William

**Affiliations:** 1 Monash University, Melbourne, Victoria, Australia

This is a clear and important article for current and future practice. The origins and development of both fields provide an interesting background to the arguments raised. The evidence at present is emerging and hopefully, this article will spur collaboration for further research. 

 The article covers many of the problems facing modern healthcare and provides a much-needed discourse into future service delivery models. More collaboration is required between rehabilitation and palliative care if we are to optimally serve the needs of our combined populations.

 I thought there would be some evidence from the MND literature about "neuro-palliative rehabilitation" - a term I have heard from our local MND specialist centre in Melbourne. I also wonder if Alex Jadad's article on the WHO definition of Health might be pertinent in the argument: “Health is the ability that we have as individuals or communities to adapt and to manage the inevitable challenges that we face through life - physical, mental, or social.” - just a thought. ^1^

Is the work clearly and accurately presented and does it cite the current literature?

Yes

If applicable, is the statistical analysis and its interpretation appropriate?

Not applicable

Are all the source data underlying the results available to ensure full reproducibility?

Yes

Is the study design appropriate and is the work technically sound?

Yes

Are the conclusions drawn adequately supported by the results?

Yes

Are sufficient details of methods and analysis provided to allow replication by others?

Yes

Reviewer Expertise:

Palliative care. Cancer Pain. End-of-Life-Care. Public Health Palliative Care.

I confirm that I have read this submission and believe that I have an appropriate level of expertise to confirm that it is of an acceptable scientific standard.

## References

[1] : How should health be defined?. *BMJ* .2008;337(dec10 1) : 10.1136/bmj.a2900 10.1136/bmj.a2900 19073663

# Peer review: Reviewer response for version 1

**DOI:** 10.21956/wellcomeopenres.18746.r44767

**Published:** 2021-07-09

**Authors:** Cathy Payne

**Affiliations:** 1 All Ireland Institute of Hospice and Palliative Care, Dublin, Ireland; 2 European Association for Palliative Care, Vilvoorde, Belgium

I think this is a very timely and well-constructed paper and would encourage all those working within both specialities to read it. The authors helpfully describe the historical and epistemological founding of both specialities before focusing on how and why they have become entwined. The assertions and sentiments of the authors that “ *effective CRPC must adapt to the highly segmented and specialized systems in which it is required to operate, recognizing that rehabilitation and palliative care are themselves co-constructors of such segmentation and specialization, but also potential agents for change*” are echoed in the work of Candy *et al.* ^1^ and Horton *et al.* ^2^ and by as they sought to characterize ways to research and effectively embed complex health interventions into practice.

 Rehabilitation delivered by palliative care services should not just be about physical function and needs to consider the broader concepts of enablement, choice and autonomy. Likewise, palliative care delivered by rehabilitation services needs to look beyond care choices as death approaches, supporting people to live as well as they can, whilst coping constructively with losses or symptoms arising from their deteriorating health.

 There is a need to recognise the social, context-sensitive, and theoretical underpinnings of combined rehabilitation and palliative care services. Not to do so will ultimately diminish the potential benefits to patients and families of this approach. I look forward to further studies and reflections on the conceptualisation of both rehabilitation within palliative care, and of palliative care within rehabilitation as these fields continue to evolve.

 The full reference for reference 46 in the paper is Dietz, J.H. 1981. Rehabilitation Oncology. UK: John Wiley and sons.

Is the work clearly and accurately presented and does it cite the current literature?

Yes

If applicable, is the statistical analysis and its interpretation appropriate?

Not applicable

Are all the source data underlying the results available to ensure full reproducibility?

Yes

Is the study design appropriate and is the work technically sound?

Yes

Are the conclusions drawn adequately supported by the results?

Yes

Are sufficient details of methods and analysis provided to allow replication by others?

Yes

Reviewer Expertise:

Working within specialist palliative care as a dietitian and an allied health professional lecturer and researcher since 2004. My PhD focused on rehabilitation in palliative care.

I confirm that I have read this submission and believe that I have an appropriate level of expertise to confirm that it is of an acceptable scientific standard.

## References

[1] : Description of complex interventions: analysis of changes in reporting in randomised trials since 2002. *Trials* .2018;19(1) : 10.1186/s13063-018-2503-0 110 10.1186/s13063-018-2503-0 29467013PMC5822627

[2] : Overcoming Challenges In Codifying And Replicating Complex Health Care Interventions. *Health Aff (Millwood)* .37(2) : 10.1377/hlthaff.2017.1161 191-197 10.1377/hlthaff.2017.1161 29401020
